# *Conus vexillum* venom induces oxidative stress in Ehrlich's ascites carcinoma cells: an insight into the mechanism of induction

**DOI:** 10.1186/1678-9199-19-10

**Published:** 2013-05-01

**Authors:** Mohamed A Abdel-Rahman, Ismail M Abdel-Nabi, Mohamed S El-Naggar, Osama A Abbas, Peter N Strong

**Affiliations:** 1 Department of Zoology, Faculty of Science, Suez Canal University, Ismailia 41522, Egypt; 2 Department of Biological Sciences, Faculty of Science, Taibah University, Madinah, KSA; 3 Department of Zoology, Faculty of Sciences, Port Said University, Port Said, Egypt; 4 Biomedical Research Center, Biosciences Division, Sheffield Hallam University, Sheffield, UK

**Keywords:** *Conus vexillum* venom, Ehrlich’s cells, Oxidative stress, Cancer, Egypt

## Abstract

**Background:**

It is estimated that venoms of marine cone snails (genus Conus) contain more than 100,000 different small peptides with a wide range of pharmacological and biological actions. Some of these peptides were developed into potential therapeutic agents and as molecular tools to understand biological functions of nervous and cardiovascular systems. In this study we examined the cytotoxic and anticancer properties of the marine vermivorous cone snail *Conus vexillum* (collected from Hurgada and Sharm El-Shaikh, Red Sea, Egypt) and suggest the possible mechanisms involved. The *in vitro* cytotoxic effects of *Conus* venom were assessed against Ehrlich’s ascites carcinoma (EAC) cells.

**Results:**

*Conus* venom treatment resulted in concentration-dependent cytotoxicity as indicated by a lactate dehydrogenase leakage assay. Apoptotic effects were measured *in vivo* by measuring levels of reactive oxygen species and oxidative defense agents in albino mice injected with EAC cells. *Conus* venom (1.25 mg/kg) induced a significant increase (*p* < 0.05) in several oxidative stress biomarkers (lipid peroxidation, protein carbonyl content and reactive nitrogen intermediates) of EAC cells after 3, 6, 9 and 12 hours of venom injection. *Conus* venom significantly reduced (*p* < 0.05) the activities of oxidative defense enzymes (catalase and superoxide dismutase) as well as the total antioxidant capacity of EAC cells, as evidenced by lowered levels of reduced glutathione.

**Conclusions:**

These results demonstrate the cytotoxic potential of *C. vexillum* venom by inducing oxidative stress mediated mechanisms in tumor cells and suggest that the venom contains novel molecules with potential anticancer activity.

## Background

It is acknowledged that natural products are one of the major sources for drug discovery and used to treat several life-threading diseases including cancer [1]. Venoms of animal species (such as snakes, scorpions, spiders and cone snails) are a combination of unique bioactive molecules that display a plethora of molecular targets and functions. Some of these molecules are currently being developed as candidate or to provide a critical template for the design of others [2].

Cancer is defined as a disturbance in mechanisms involved in the control of cell growth and division [3,4]. It is well known that resistance to apoptotic signals is one of the characteristic features of cancer cells [5]. Chemotherapy is the usage of conventional antitumor drugs to treat several types of cancerous cells. While chemotherapy can be quite effective in treating advanced or metastatic diseases, it often accompanied with many harmful side-effects through inducing severe damage to normal cells and tissues [6-8]. Moreover through particular cellular and molecular changes, cancer cells eventually become not susceptible to chemotherapeutic agents. These changes include up-regulation the enzymes of drug detoxification and drug transporters and alteration in the molecular targets of drugs. Also, drug tolerance may be due to increase the ability of cancer cells to evade apoptosis through suppression apoptotic pathways and improve the machinery of DNA repair [9-11]. Thus, development of alternative anticancer drugs with minimal side-effects is urgently required.

The scientific literature on the antitumor activities of snake venoms extends back many decades [12,13]. More recently, peptides with specific antitumor activity have been isolated, such as cystatin [14]. However the high toxicity of snake venoms often halts their therapeutic properties and in most cases, the cytotoxic doses for malignant and normal cells are the same. In addition to snake venom, anti-cancer efficacy of bee venom has been reported using both in vitro and in vivo approaches [15,16]. Bee venom has been reported to be cytotoxic and induce apoptosis in various tumour cell lines [17,18]. The promotion of apoptosis through several cancer cell death mechanisms is essential for bee venom-induced anticancer effects (and the bee venom peptide, melittin) [19]. Scorpion venom also possess the property of inhibiting growth of various types of cancers [20]. Several cytotoxic antitumor peptides have been characterized from scorpion venom, which exert their action by distinct mechanisms: as an ion channel blocker, as a matrix metalloproteinase (MMP-2) inhibitor and as an apoptosis inducer, activating intracellular pathways [21-23].

The venoms of cone snails, a family of widely distributed marine mollusks, contain a large number of small peptides (conotoxins and conopeptides) which have evolved separately in approximately 700 species of specific fish, mollusk and worm-hunting animals [24]. It is estimated that more than 50,000 conopeptides exist, but less than 0.1% have been functionally characterized. Those that have been examined are specific for an amazingly diverse set of important pharmacological targets, including voltage gated ion channels (Na, K, Ca), neurotransmitter receptors (acetylcholine, glutamate) and transporters (noradrenaline) [2]. Their small size, relative ease of synthesis and structural stability also make them important templates for the design of novel drugs and indeed several are presently in clinical trials [25].

We have recently demonstrated that crude venom of the vermivorous snail *C. vexillum* caused an array of cytotoxic effects in mammalian systems which were attributed to the venom’s ability to induce oxidative stress [26]. Venoms collected from different locations in the Red Sea (Hurgada and Sharm El-Shaikh) also showed clear differences in venom potency. The present study was designed to extend our earlier work, by examining the cytotoxic activity and mechanism of action of *C. vexillum* venom on EAC cells, both *in vitro* and *in vivo*. To the best of our knowledge, this is the first study of its kind on this *Conus* species.

## Methods

### Venom preparation

Specimens of *C. vexillum* were collected from two different sites (Hurgada and Sharm El-Shaikh) in the Red Sea, Egypt [26]. Venom glands were dissected as described previously by Cruz *et al*. [27] and crude venom from each location was extracted and lyophilized [26]. Pooled venoms were stored at −80°C until further use.

### Experimental animals and ascites tumor

All animal procedures and experimental protocols were approved by the Research Ethics Committee of Suez Canal University and were carried out in accordance with the *Guide for the Care and Use of Laboratory Animals* (http://www.nap.edu/catalog/12910.html). Adult male Swiss albino mice weighing 20–25 g were purchased from the breeding unit of Theodor Bilharz Research Institute (Giza, Egypt). The animals were maintained under controlled conditions of temperature, humidity and on a 12 hour-light/dark cycle, with free access to standard pellet diet and water. The first inoculum of EAC cell line was purchased from the Department of Tumor Biology, National Cancer Institute, Cairo University. The cell line was inoculated by serial intraperitoneal (IP) passages of 10^6^ cells per mouse. Cells were grown in the peritoneal cavity of mice and transferred every ten days to new animals. Mice were monitored daily for signs of tumor progression, including the amount of abdominal distension and signs of illness and distress. The volume of ascites fluid was determined by needle (18–22 gauge) aspiration. Withdrawal of ascites fluid was performed under aseptic conditions.

### Isolation of EAC cells

Based on the method described by Mookerjee et. al. [28] peritoneal fluid containing the tumor cells was withdrawn, collected in sterile petri plates and incubated at 37°C for two hours. The cells of macrophage lineage adhered to the bottom of the petri dishes. Since EAC cells do not adhere to synthetic surfaces *in vitro*, the non-adherent cell population was gently aspirated and washed repeatedly with phosphate buffered saline [29]. More than 93% of the non-adherent cells were morphologically characterized as EAC cells by Wright staining [30] and viability was assessed by Trypan Blue dye exclusion [31].

### Lactate dehydrogenase (LDH) assay

EAC cells (1 × 10^6^ cells/mL) were incubated in a 96-well plate with varying concentrations (10, 20 and 30 μg/mL) of *C. vexillum* venom for one hour at 37°C. LDH released into the medium was determined spectrophotometrically (340 nm) by measuring the rate of decrease of NAPDH, using commercial kit (Spinreact, Sant Esteve de Bas, Spain). Data was expressed as units/liter (U/L).

### Antitumor activity of *Conus* venom

The antitumor activity of *C. vexillum* venom from each geographical location was evaluated. Fifty-four mice were inoculated with the EAC cells as previously mentioned. After successful establishment of the tumor (ten days after tumor inoculation), mice were randomly assorted into three groups. A Hurgada-treated group (24 animals) and a Sharm El-Shaikh-treated group (24 animals) were both injected IP with a sublethal dose of *C. vexillum* venom (1.25 mg/kg) [26]*.* A third control group (six animals) was injected with 100 μL of sterile saline.

Ascites fluid containing EAC cells was drawn from the peritoneal cavity of treated and control groups after 3, 6, 9 and 12 hours of venom administration. The following biochemical parameters were measured in the EAC cells of treated and control groups: the level of lipid peroxidation (LPx) and protein carbonyl contents (PCC), glutathione (reduced form) content (GSH), the activities of Cu/Zn-superoxide dismutase (Cu/Zn-SOD), catalase (CAT) and total antioxidant capacity (TAC). The level of nitric oxide (NO) was measured in the supernatant of ascites fluid.

### Malondialehyde (MDA) assay

The extent of lipid peroxidation in EAC cells was determined by measuring the production of malondialdehyde, an indicator of oxidative damage and oxidative stress [31]. Malondialdehyde was determined by incubating samples with thiobarbituric acid and measuring reaction products at 532 nm [32]. 1,1,3,3-tetramethoxypropane [malonaldehyde bis(dimethyl acetal), Sigma-Aldrich, USA] was used as an external standard and the results were expressed as μmoles of MDA/mg.

### Protein carbonyl content (PCC)

PCC was quantified using dinitrophenylhydrazide (DNPH) [33]. Protein was precipitated with an equal volume of 1% trichloroacetic acid (TCA) and the pellet was resuspended in 1 mL of 2,4- dinitrophenylhydrazide (Sigma-Aldrich, USA), 10 mM, dissolved in 2 N HCl. Based on the method described by Fulle *et al.*[34], separate blanks were prepared by adding 1 mL of 2 N HCl without DNPH. Samples were left at room temperature for one hour in the dark and vortexed every 15 minutes. An equal volume of 20% TCA was added and after centrifugation (12,000 × g, 15 minutes, 4°C), pellets were washed (three times) with 1 mL of ethanol: ethyl acetate (1:1) to remove free DNPH and lipid contaminants. The final pellet was dissolved in 6 M guanidine (1 mL, 1 hour, 37°C shaking water bath). The solution was centrifuged (12,000 × g, 15 minutes) and the carbonyl content (nmol/mg), measured as protein phenylhydrazone derivatives, was determined at 370 nm using an absorption coefficient of 22,000 M^−1^ Cm^1^.

### Estimation of nitric oxide (NO)

Nitric oxide was measured in the supernatant of ascites fluid by a spectrophotometric method based on the Griess reaction [35]. One hundred microliters of supernatant was mixed with an equal volume of Griess reagent (one part 0.1% naphthylethylendiamine dihydrochloride in distilled water plus one part 1% sulfanilamide in 5% concentrated H_3_PO_4_), at room temperature for ten minutes. The absorbance was measured at 540 nm. Sodium nitrite was used as a standard.

### Reduced glutathione (GSH)

The content of glutathione in EAC cells of control and treated groups was estimated according to Beutler *et al*. [36]. Aliquots of 0.2 mL of ascites fluid were added to 1.8 mL distilled water and 3 mL of precipitating solution (1.67 gm glacial metaphosphoric acid, 0.2 gm EDTA and 30 gm NaCl in 100 mL distilled water) and the mixture was centrifuged (2200 × g, 15 minutes, 4°C). To 1 mL of supernatant it was added sequentially, sodium dihydrogen phosphate (4 mL, 0.3 M) and DTNB reagent [0.5 mL, 40 mg 5,5` dithiobis-2-nitrobenzoic acid (Sigma-Aldrich, USA) dissolved in 100 mL 1% sodium citrate] and the absorbance was measured at 412 nm. Reduced glutathione (Sigma-Aldrich, USA) was used as standard.

### Cu/Zn-superoxide dismutase (Cu/Zn-SOD) and catalase (CAT)

Superoxide dismutase (Cu/Zn SOD; EC 1.15.1.1) activity was estimated in the lysate of EAC cells according to Misra and Fridovich [37]. The rate of inhibition of auto oxidation was monitored at 560 nm; the amount of enzyme required to produce 50% inhibition is defined as one unit of enzyme activity. SOD activity was expressed as units/mL. Catalase (CAT; EC 1.11.1.6) activity was determined using the method of Aebi [38]. Samples were prepared in phosphate buffer (50 mM, pH 7) and Triton-X 100 (1%, v/v) was added to increase the observable CAT activity by releasing the enzyme from peroxisomes [39]. CAT was measured by monitoring the decomposition of H_2_O_2_ at 240 nm. The enzyme activity was calculated using a molar extinction coefficient of 43.6 mol.

### Total antioxidant capacity (TAC)

The total antioxidant capacity in EAC cell lysates was determined according to Koracevic *et al*. [40]. As in our previous work [26], the ability of antioxidants to inhibit the H_2_O_2_-induced oxidation of 2,2’-azinobis (3-ethylbenzothiazoline-6-sulphonate; ABTS) was measured spectrophotometrically by the reduction in concentration of the cation radical ABTS^+^, absorbing at 600 nm and expressed as mmol/L.

### Statistical analysis

SPSS® statistical software (v. 17.01 SPSS Inc., USA) was used in all data analyses [41]. Descriptive analyses including mean and standard error were applied to all biochemical measurements. Differences in the effects of *Conus* venom between control and treated groups were assessed using the Student's unpaired *t*-test [42]. One-way analysis of variance (ANOVA) followed by a Dunnett *post hoc* test was performed to evaluate eventual significant differences in the biochemical measurements between control and treated groups.

## Results

### *In vitro* cytotoxicity of *Conus* venom

The membrane integrity of EAC cells treated with *C. vexillum* venom, obtained from either Hurgada or Sharm El-Shaikh, was evaluated by measuring the levels of LDH activity in cell culture supernatants. LDH levels in cells incubated with *Conus* venom for one hour from each location showed a significant (*p* < 0.05) dose-dependent increase in comparison to the levels in vehicle-treated cells (Table 1). At doses of 10, 20 and 30 μg/mL of Hurgada venom, release of LDH increased to 48.9, 131.7 and 196.7% respectively, with respect to the control. For Sharm El-Shaikh venom, analogous values were 43.6, 107.5 and 171.9%, with respect to the control. Over the course of the experiment, Hurgada venom was more potent than Sharm El-Shaikh venom in increasing LDH levels and was statistically significant (*p* < 0.05) at ≥ 20 μg/mL venom.

**Table 1:** **Changes in LDH levels of EAC cells incubated with *Conus vexillum *venom collected from Hurgada and Sharm El-Shaikh (Red Sea, Egypt)**

**Venom concentration (μg/mL)**	**LDH Activity (U/L)**		
	**Control**	**Hurgada**	**Sharm El-Shaikh**
10	295.53 ± 8.1^a^	439.98 ± 10.6^*^ (+48.9)	424.38 ± 9.0^*^ (+43.6)
20		684.58 ± 8.3^*^ (+131.7)	613.18 ± 8.7^*,#^ (+107.5)
30		875.00 ± 12.1^*^ (+196.7)	803.6 ± 11.5^*,#^ (+171.9)

^a^Values are presented as mean ± SE (n = 5 per group). Values between brackets represent percent of change. *Significant difference between control (EAC control group) and treated group using Student’s unpaired *t*-test (*p* < 0.05). ^#^Represents a significant difference between Hurgada and Sharm El-Shaikh groups using Student’s unpaired *t*-test (*p* < 0.05).

### *In vivo* cytotoxic effects of *Conus* venom on EAC cells

**Figure 1:**
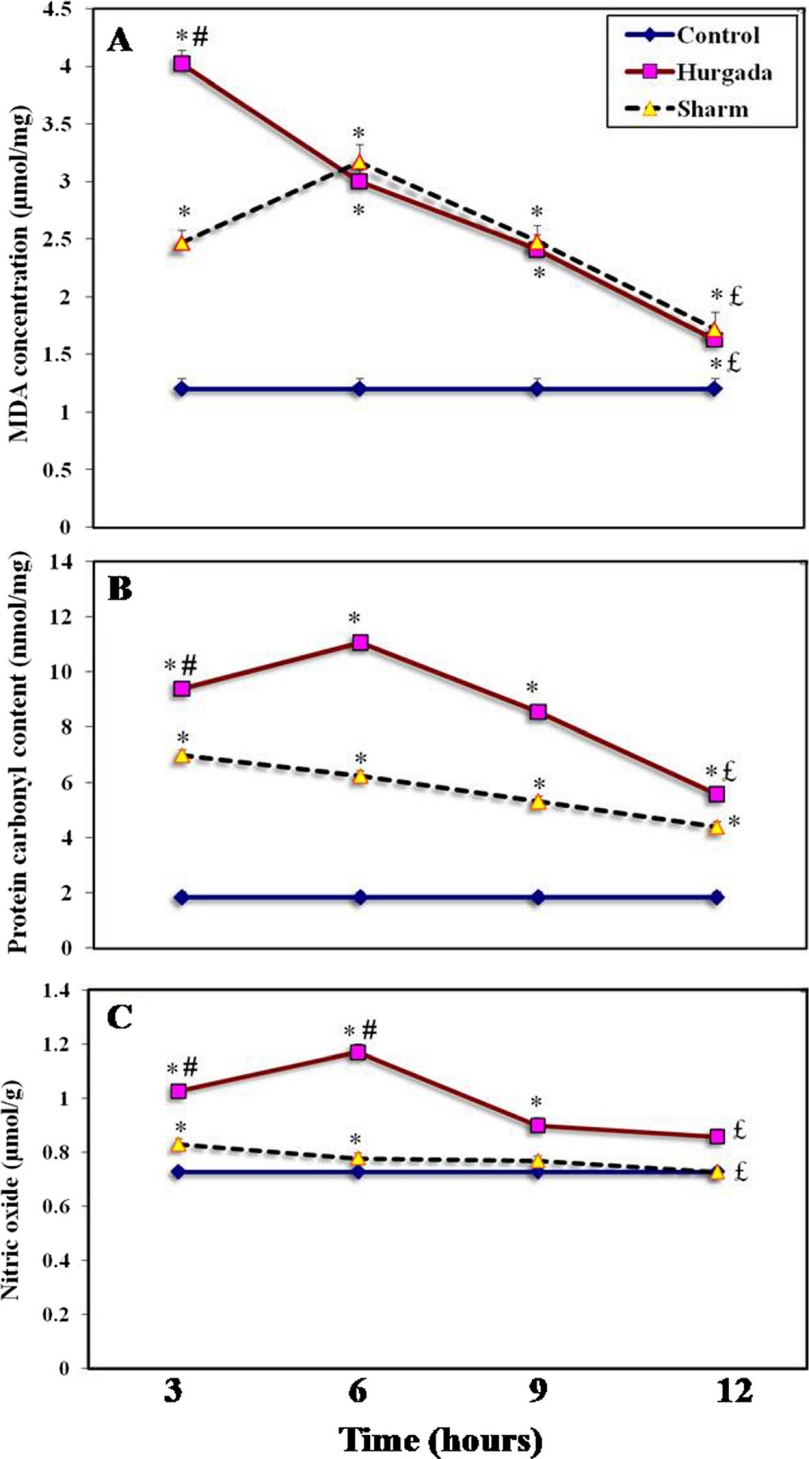
*Conus vexillum *venom (1.25 mg/kg) collected from Hurgada and Sharm El-Shaikh induced intracellular oxidative stress in EAC cells assessed by the level of (A) lipid peroxidation, (B) protein carbonyl content and (C) level of nitric oxide.^*^Significant difference between control (EAC control group) and treated group using Student’s unpaired *t*-test (*p* < 0.05). ^#^Represents a significant difference between Hurgada and Sharm El-Shaikh groups using Student’s unpaired *t*-test (*p* < 0.05). ^£^Significant difference between treated groups using one-way ANOVA (*p* < 0.05).

There are several assays available to measure oxidative stress. One such indicator is the extent of lipid peroxidation (LPx) as measured by thiobarbituric acid derivatives of key metabolic markers such as malondialdehyde (MDA). Protein carbonylation (PCC) is generally recognized as a key step in the production of oxidized proteins and the conversion of NO to nitrite and nitrate is typical metabolic event in oxygenated solutions. The results in Figure 1 illustrate the effects of injection of *C. vexillum* venom (1.25 mg/kg) from each location into tumor-bearing mice, on the oxidative stress biomarkers (LPx, PCC and NO) at different time intervals (3, 6, 9 and 12 hours).

It was observed that LPx (as measured by MDA concentration) was significantly increased (*p* < 0.05) at all time intervals in both Hurgada and Sharm El-Shaikh venoms with respect to control groups (Figure 1 – A). However, levels of MDA increased more rapidly after injection of Hurgada venom (up to 235% increase over control levels after three hours); injection of Sharm El-Shaikh venom took twice as long to produce increased levels of MDA (up to 164% after six hours) which were considerably lower than that produced by Hurgada venom. One-way ANOVA revealed that differences in the concentration of LPx products between treated groups of Sharm El-Shaikh venoms was not as great (F_1,3_ = 35.86, *p* < 0.001) as Hurgada venoms (F_1,3_ = 111.69, *p* < 0.001). Analysis of LPx products revealed significant effects for venom (F_2,72_ =13.01, *p* < 0.05), time (F_3,72_ = 71.80, *p* < 0.001) and the interaction of venom and time (F_6,72_ = 24.16, *p <* 0.001) using two-way ANOVA.

The PCCs of EAC cells from mice injected with both venom groups were significantly increased (*p* < 0.05) at all times intervals compared with control values (Figure 1 – B). On average, Hurgada venom was approximately 1.4 times as potent as Sharm El-Shaikh venom in inducing protein oxidation. Using one-way ANOVA, significant differences in PCCs were detected between the treated groups of both Hurgada and Sharm El-Shaikh (F_1,3_ = 470.04, *p* < 0.001 and F_1,3_ = 221.80, *p* < 0.001, respectively). According to the *post hoc* comparisons, six hours of Hurgada venom treatment produced the maximum increase (+ 501%) in protein oxidation relative to control. Using two-way ANOVA, PCCs revealed significant effects for venom (F_2,72_ = 663.60, *p* < 0.001), time (F_3,72_ = 214.33, *p* < 0.001) and the interaction of venom and time (F_6,72_ = 45.54, *p* < 0.001). *Conus* venom enhanced production of NO in Hurgada venom-injected animals (Figure 1 – C) and by six hours there was a 61% increase in levels of NO with respect to controls (*p* < 0.05). In comparison, injection of Sharm El-Shaikh venom had a negligible effect (maximum 7%) on NO levels. Differences between Hurgada and Sharm El-Shaikh were statistically significant (*p* < 0.05) up to six hours post injection. Statistical analysis of NO levels revealed significant effects for venom (F_2,72_ = 491.74, *p* < 0.001), time (F_3,72_ = 77.17, *p* < 0.001) and the interaction of venom and time (F_6,72_ = 42.09, *p* < 0.001).

**Figure 2:**
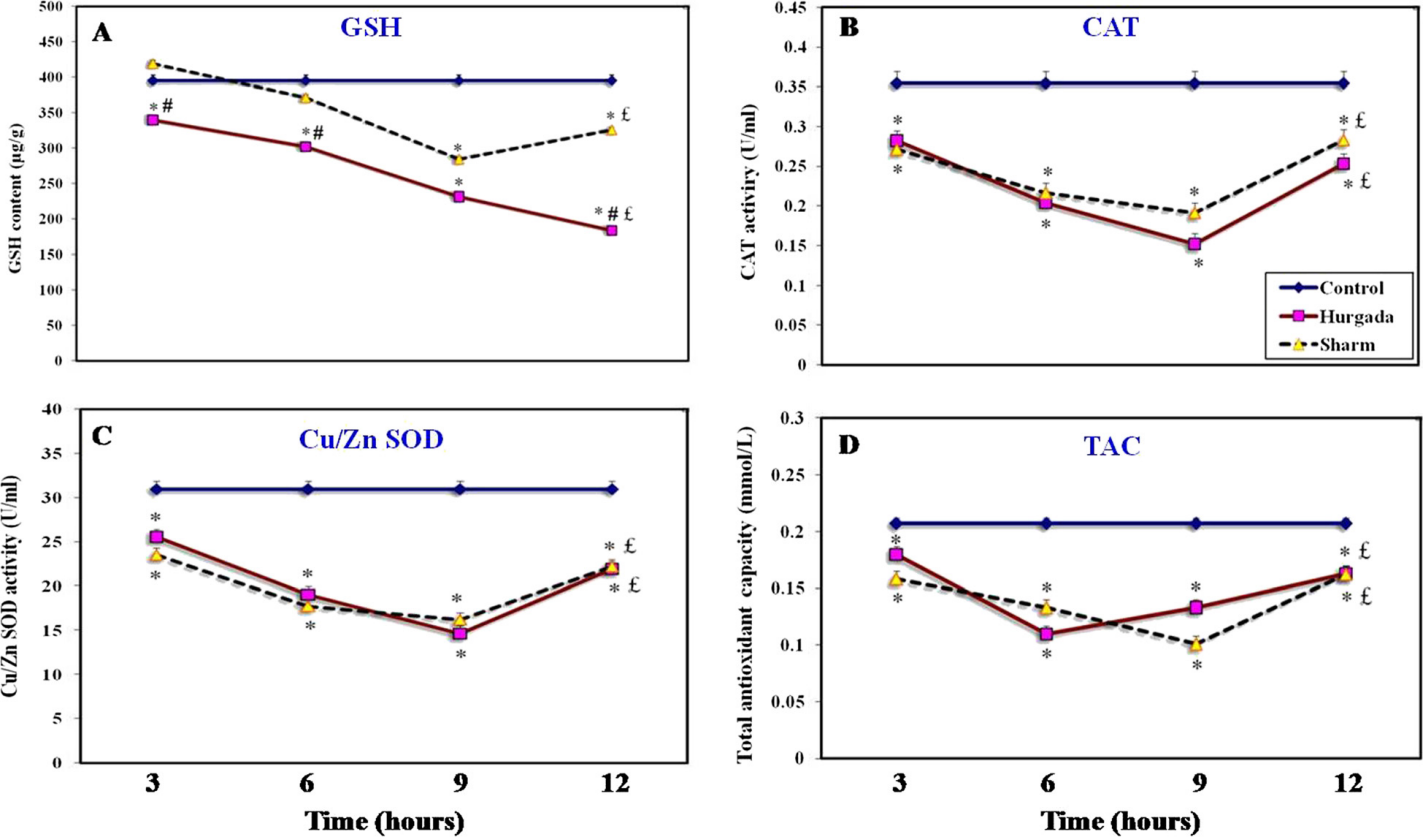
**Changes in the antioxidant measurements GSH (panel A), CAT (panel B), Cu/Zn SOD (panel C) and TAC (panel D) of EAC cells after administration of *Conus vexillum *venom (1.25 mg/kg) collected from Hurgada and Sharm El-Shaikh.**

The *in vivo* effects of injecting *Conus* venom into EAC-tumor-bearing mice, on the activities of representative oxidative defense enzymes (CAT and Cu/Zn-SOD) was examined, together with the effects on cellular GSH (as a non-enzymatic antioxidant) and the total antioxidant capacity (TAC). The level of cellular GSH was significantly decreased (*p* < 0.05) at all time intervals in mice injected with Hurgada venom, continuing to decline to 46% of control levels at 12 hours (Figure 2 – A). By comparison, in mice injected with Sharm El-Shaikh venom, the levels of GSH declined at a slower rate and began to recover after nine hours (Figure 2 – A). Using bivariate analysis of covariance, cellular GSH brought significant effects for venom (F_2,72_ = 81.80, *p* < 0.001), time (F_3,72_ = 42.00, *p* < 0.001) and the interaction of venom and time (F_6,72_ = 4.27, *p* < 0.05).

There was a significant decrease (*p* < 0.05) in the activities of both CAT and Cu/Zn-SOD in mice injected with either Hurgada and Sharm El-Shaikh venoms at all time intervals as compared with the control groups. The pattern of CAT and SOD levels was the same in both Hurgada and Sharm El-Shaikh venoms, declining to a maximum of approx 56% and 47% respectively in the two locations, at nine hours (Figure 2 – B and C). Interestingly, GSH levels at the different time points mirrored CAT and Cu/Zn SOD levels in the case of Sharm El-Shaikh venom, with a partial recovery after nine hours. Unpaired *t*-tests showed significant decreases in the level of TAC in EAC cells from both venom groups at all time intervals as compared with the control group (*p* < 0.05). In distinction to the other antioxidant markers, there was a significant decrease in TAC levels in mice injected with Hurgada venom, in the second time period (47% decline from control levels at six hours). In comparison, TAC levels in mice injected with Sharm El-Shaikh venom declined more slowly, although the eventual amount of decline was very similar (51% decline from control levels after nine hours) (Figure 2 – D). Moreover, the activities of oxidative defense enzymes (CAT and SOD) and TAC showed significant effects for venom (F_2,72_ = 4.09, 0.34 and 2.61, *p* < 0.05), time (F_3,72_ = 33.1, 42.5 and 30.1, *p* < 0.001) and the interaction of venom and time (F_6,72_ = 1.69, 1.7 and 6.1, *p* < 0.05), respectively.

## Discussion

Venoms from marine cone snails (*Conus*) have received much attention over the last few decades due to their extraordinary complexity and diversity [43]. Each *Conus* species synthesizes its own characteristic repertoire of ∼ 1100-1900 toxin peptides, and it has been estimated that the toxin library of the entire cone snail genus comprises as many as 500,000 different bioactive compounds [24,44]. The vast majority of these peptides have been shown to target various types of ion channels, both ligand-gated and voltage-gated [45]. In comparison, there are only a few reports describing the cytotoxic actions of *Conus* venoms on eukaryotic cells [26,46,47].

The first cytolytic peptide (conolysin-Mt) was isolated from the vermivorous cone snail venom *Conus mustelinus*[47]. The characterization of conolysin expanded the known repertoire of conopeptide mechanisms to include membrane perturbation. Cytolytic peptides, defined by their ability to partially or completely destroy cell membranes, are among the largest group of toxins produced by living organisms, which include bacteria, viruses, insects, scorpions, spiders, reptiles, and marine invertebrates [48]. By targeting the lipid bilayer of the cell membrane, cytolytic peptides can affect a wide range of biological processes. In our study, an attempt has been made to elucidate the cytotoxic potential of *Conus* venom (collected from Hurgada and Sharm El-Shaikh) and its underlying mechanism of action, using EAC cells.

The cytotoxic potential of *Conus* venom from these two geographical sites has been evaluated *in vitro* against EAC cells by measuring LDH activity. It is well documented that the *in vitro* release of LDH provides an accurate measure of cell membrane integrity and cell viability. This assay is based on the release of the cytosolic enzyme LDH from cells, which catalyses the conversion of lactate to pyruvate and cannot be detected extracellularly unless cell damage has occurred. The LDH levels in EAC cells incubated with *Conus* venom for one hour showed a significant (*p* < 0.01) dose-dependent increase in comparison to the levels in vehicle-treated cells. The release of LDH indicates a loss of cell membrane integrity and is therefore an indirect method to assess the venom-induced cytotoxicity [20,49]. Abdel-Rahman *et al*. [26] attributed the direct cytotoxic effects of *C. vexillum* on mammalian cells to the presence of proteolytic enzymes in the crude venom. Proteolytic enzymes in *Conus* venom are primarily responsible for venom-induced necrotic activity and could be injected by cone snails to elicit proteolytic degradation of the extracellular matrix in the prey [46]. Indeed, Cathepsin D and kallikrein-like proteins have recently been identified in the venom glands of *C. victoriae* and *C. novaehollandiae,* respectively [43]. Cathepsin D (aspartyl protease) and kallikrein (trypsin and serine proteases) have also been found in other animal venoms [50-52].

Interestingly, our toxicological data revealed significant differences in the efficacy of *C. vexillum* venom collected from the two Egyptian locations, Hurgada and Sharm El-Shaikh. The data showed that the venom obtained from Hurgada was more potent than that obtained from Sharm El-Shaikh. We have attributed the difference in venom potency to variation in the expression of peptides from *C. vexillum* collected from these two locations [26]. For example, the presence of the m/z peptide 2924.7 (calcium channel blocker) in the venom of Sharm El-Shaikh may explain why venom-induced oxidative stress was significantly higher in the case of Hurgada venom. The presence of this calcium channel blocker in Sharm El-Shaikh venom delays rises in intracellular calcium concentrations in venom-treated cells and hence slows the elevation of oxidative stress markers in comparison with Hurgada venom [26].

It is well documented that oxidative stress is extremely deleterious to cells and reactive oxygen species (ROS) are engaged in the etiology and progression of several diseases including cancer. Several environmental oxidants such as heat shock, UV irradiation, infections, and toxins [26,53,54] can induce oxidative stress that shifting the cellular redox status to a more oxidized state. In normal conditions, enzymatic and non-enzymatic antioxidants are capable of neutralizing harmful effects of ROS and protect cellular components from damage. However, under conditions of excessive oxidative stress, ROS can damage cellular components and interfere with critical cellular activities [55]. Numerous studies have provided evidence that ROS are directly involved in oxidative damage with cellular macromolecules such as lipids, proteins and nucleic acids, leading to cell death [56]. Cancer cells have active protective mechanisms to prevent lipid peroxidation. It has been demonstrated that the presence of relatively low levels of the NADPH-cytochrome-P450 electron transport chain may explain the remarkable decrease of lipid peroxidation in cancer cells when compared to normal cells [57].

In order to examine *Conus* venom induced oxidative stress, the changes of intracellular ROS of EAC cells were measured. *Conus* venom administration (1.25 mg/kg) to tumor-bearing albino mice induced significant increases in the oxidative stress biomarkers (MDA, PCC) of the propagated EAC cells. We have recently suggested possible mechanisms by which the venom of *C. vexillum* induces cellular oxidative damage in murine cells [26]. Damage could be attributed to phospholipase A_2_ activity – for example an enzyme analogous to conodipine-M – on cell membranes, causing the release of arachidonic acid [58]. Arachidonic acid can potentiate cell damage by converting apoptosis to necrosis through lipid peroxidation and the promotion of DNA fragmentation [59].

NO may also play a prominent role in cytotoxicity induced by *Conus* venom. Our results showed that *Conus* venom significantly increased the production of NO in the ascites fluid of treated animals, especially in Hurgada venom. The elevation of NO levels may be due to the activation of macrophages present in the ascites fluid. It has been found that macrophages play an important role against neoplastic cells [60]. Activated macrophages initiate cell death programs through the release of soluble immune mediators [61]. One mechanism that macrophages use to exert their cytolytic effects on target tumors is by the release of NO through activation of NADPH-dependent NO synthase [62,63]. NO can cause growth arrest and apoptosis because of its high reactivity with iron-and thiol-containing macromolecules, thereby inhibiting enzymes of the TCA cycle and those involved in mitochondrial respiration, as well as DNA synthesis and repair [64,65]. It can be noted that our data revealed that increases in NO levels paralleled a significant increase in the level of PCC, which is an indication of protein damage of EAC cells.

Several previous studies have clarified the influence of ROS overproduction on intracellular Ca^2+^ levels. Chandra *et al*. [66] found that increases in the concentrations of intracellular ROS were a sign of the onset of apoptotic processes; they cited ROS production as a critical determinant of the toxicity associated with exposure to chemotherapeutic drugs. It has been shown that oxidative stress increases intracellular Ca^2+^ concentrations, leading to activation of endonucleases which degrade DNA and, ultimately, contribute to cell damage [67]. Orrenius *et al*. [68] found that free radical overproduction may inhibit Ca^2+^ ATPases and this leads to altered regulation of Ca^2+^ levels and cell death. Ermak *et al.*[53] clearly demonstrated the influence of rising intracellular Ca^2+^ concentration induced by oxidative stress. They found that oxidative stress increases the process of Ca^2+^ diffusion from both the endoplasmic reticulum and extracellular environment into the cytoplasm. Consequently, the elevation of cytoplasmic Ca^2+^ concentrations causes Ca^2+^ influx into both mitochondria and nuclei. High concentration of Ca^2+^ in mitochondria leads to cell damage through disruption normal cellular metabolism. In nuclei, high Ca^2+^ levels modulate transcription factors and nucleases that regulate cell apoptosis. Moreover, the enzymes (e.g. nitric oxide synthase) that produce free radicals can be activated by the elevation of Ca^2+^ level [69]. Therefore, by increasing cytoplasmic Ca^2+^ concentrations, oxidants can also indirectly cause more oxidant production and further raise Ca^2+^ levels.

In the present work, increased levels of cellular oxidative stress in EAC cells treated with *Conus* venom were also accompanied by a remarkable decrease in the levels of antioxidants in treated cells. The results clearly demonstrated that *Conus* venom significantly reduced the content of GSH of tumor cells. Modifications of GSH metabolism have been postulated as being useful in cancer therapy [70]. Indeed, the elevation of intracellular GSH content has been associated with mitogenic stimulation [71,72]. GSH has been suggested as a potential regulator of protein synthesis, DNA synthesis and cell proliferation [72,73]. Several previous in vivo and in vitro studies have revealed that the amino acid precursors (glutamine and methionine) for GSH synthesis play a crucial role in cancer metabolism. Tumour cells use the amino acid glutamine as a main respiratory fuel [74] and methionine in cell growth [75]. Moreover, our data showed that the depletion of GSH content was concomitant with a reduction in the activity of enzymatic antioxidants (CAT and SOD) as well as a reduction in the level of TAC. The observed reduction in the level of antioxidants of tumor cells from venom-treated animals may explain the role of oxidative stress in cell damage. It is well established that antioxidant defense enzymes such as SOD and CAT play a crucial role in maintaining cellular homeostasis by detoxifying the generated ROS such as superoxide radicals and H_2_O_2_[76]. Our findings are corroborate those of Sun *et al*. [77], who reported that the inhibition of cell growth and apoptotic activity of the snake venom fraction OHAP-1 (*Okinawa Habu* apoxin protein-1) was reversed by the addition of GSH and catalase to rat and human malignant glioma cell lines.

## Conclusions

*Conus* venoms have provided extensive libraries of toxins active against a diverse range of ion channel proteins, belonging to both voltage-gated and neurotransmitter-activated gene families. The recognition that cone snails also have cytolytic peptides in their venom arsenals should not be surprising, given that similarly acting peptides in snake venom have been known for thirty years but it was only a few years ago that the first *Conus* venom cytolytic peptide, conolysin MT was discovered [47]. Our present study aims at contributing to the study of the mechanism of action of these cytolytic toxins from *Cone* snails that interact with and disrupt cell membranes. Our data indicate that *C. vexillum* venom contains components which induce oxidative stress mechanisms in Erhlich’s ascites carcninoma cells and suggest that the venom contains novel molecules with potential anticancer activity.

### Ethics committee approval

The present study was approved by the Research Ethics Committee of Suez Canal University and was carried out in accordance with the *Guide for the Care and Use of Laboratory Animals* (by the Committee for the Update of the Guide for the Care and Use of Laboratory Animals of the National Research Council, Washington, USA, 2011).

## Competing interests

The authors declare no conflicts of interest.

## Authors’ contributions

MAA designed and supervised the entire project, participated in analyzing the data and wrote the initial draft of the manuscript. IMA reviewed the manuscript. MSE carried out biochemical experiments and participated in analyzing the data. OAA reviewed the manuscript. PNS reviewed the manuscript and participated in its coordination. All authors read and approved the final manuscript.

## Financial source

This project has been financially supported by the Sector of Post-graduate Studies and Researches Affairs, Suez Canal University (Ismailia, Egypt).
